# Prognostic impact of adenomyosis in cervical cancer: insights from machine learning–driven survival analysis

**DOI:** 10.1590/1806-9282.20251656

**Published:** 2026-06-29

**Authors:** Cansu Önal Kanbaş, Esra Keles, Esma Özacar Mert, Cansu Ergenç Özdaş, Hatice Kübra Can

**Affiliations:** 1 University of Health Sciences, Kartal Dr. Lütfi Kırdar City Hospital, Department of Obstetrics and Gynecology – Istanbul, Turkey.; 2 University of Health Sciences, Kartal Dr. Lütfi Kırdar City Hospital, Department of Gynecologic Oncology – Istanbul, Turkey.; 3 Ankara Yildirim Beyazit University, School of Business, Department of Finance and Banking – Ankara, Turkey.; 4 Kelkit State Hospital, Department of Obstetrics and Gynecology – Gümüşhane, Turkey.

**Keywords:** Uterine cervical neoplasms, Adenomyosis, Prognosis, Survival analysis, Machine learning

## Abstract

**OBJECTIVE:**

The aim of this study was to determine whether adenomyosis is an independent prognostic factor in cervical cancer using integrated survival analysis and machine-learning models.

**METHODS:**

This retrospective cohort study included 131 patients with early-stage cervical cancer treated surgically between 2008 and 2020. Patients were stratified by the presence (n=28) or absence (n=103) of adenomyosis based on final histopathology. Kaplan-Meier curves and log-rank tests assessed overall survival. Independent prognostic factors were identified through multivariate Cox regression and logistic regression analyses, supplemented by machine-learning decision tree modeling to evaluate variable importance and model performance.

**RESULTS:**

Women with adenomyosis had no significant differences in tumor size, histology, depth of stromal invasion, lymphovascular space invasion, parametrial involvement, lymph node metastasis, or International Federation of Gynecology and Obstetrics 2018 stage. Kaplan-Meier analysis demonstrated shorter overall survival in the adenomyosis group (median overall survival 31.3 vs. 64.8 months, log-rank p=0.045). Multivariate Cox regression identified age, tumor size, International Federation of Gynecology and Obstetrics stage III, histologic grade, lymphovascular space invasion, parametrial infiltration, and vaginal involvement as independent determinants of overall survival (all p<0.05). Adenomyosis status did not retain prognostic significance after adjustment (HR 0.91, p=0.818). Decision tree models corroborated these findings, with International Federation of Gynecology and Obstetrics stage and tumor size emerging as the most influential predictors of survival and recurrence.

**CONCLUSION:**

Although adenomyosis was associated with shorter overall survival in univariate analysis, it did not function as an independent prognostic factor after adjustment for established clinicopathological variables in both multivariate Cox regression and machine-learning decision tree models. These findings indicate that prognostic stratification in cervical cancer should remain guided by tumor burden, stage, and histopathological risk factors.

## INTRODUCTION

Cervical cancer (CC) remains a major global health challenge, ranking as the fourth most common malignancy in women worldwide, with an estimated 604,000 new cases and 342,000 deaths annually. Despite the widespread implementation of screening programs and the introduction of prophylactic human papillomavirus (HPV) vaccination, incidence and mortality rates remain disproportionately high in low- and middle-income countries, where access to preventive and therapeutic resources is limited^1,2^. Although persistent infection with high-risk HPV constitutes the necessary etiological agent, the clinical trajectory of CC is shaped by a complex interplay of host genetic susceptibility, immune competence, and coexisting gynecological conditions, underscoring the need for continued refinement of prognostic stratification beyond established staging systems^3^.

A frequent clinical observation in gynecologic oncology is the coexistence of benign uterine conditions with malignant disease. Adenomyosis (AM), characterized by the ectopic infiltration of endometrial glands and stroma into the myometrium, is a prevalent benign condition identified in approximately 20–35% of hysterectomy specimens, with reported prevalence varying substantially depending on diagnostic criteria and histopathological sampling protocols^4^. The prognostic relevance of AM has been most extensively investigated in the context of endometrial carcinoma (EC), where the evidence remains conflicting. Several studies and meta-analyses have reported associations between coexistent AM and earlier stage, lower histologic grade, and improved overall survival (OS), whereas others have found no significant independent prognostic effect or have even identified correlations with more aggressive pathological features^5,6^. These discrepancies likely reflect differences in study design, sample size, AM diagnostic criteria, and the adequacy of adjustment for confounding variables.

In contrast, the relationship between AM and CC has received far less scholarly attention, and the existing evidence is sparse and methodologically heterogeneous. Yang et al. reported that patients with CC and coexistent AM exhibited less aggressive tumor behavior, including reduced deep stromal invasion and vaginal infiltration, yet this did not translate into demonstrably improved survival outcomes^7^. The paucity of studies, the typically small AM cohorts within CC populations, and the absence of rigorous multivariate adjustment in many prior analyses leave a significant knowledge gap regarding whether AM exerts a clinically meaningful influence on CC prognosis. Clarifying this relationship carries practical importance: if AM were confirmed as an independent prognostic factor, it could influence risk stratification, treatment planning, and patient counseling; conversely, establishing that it is not independently prognostic would prevent unnecessary clinical concern when AM is incidentally identified in CC hysterectomy specimens.

Therefore, the present study was designed to systematically evaluate the clinicopathological features and survival outcomes of patients with CC, stratified by the presence or absence of AM. To provide a methodologically robust assessment, we employed a multi-method analytical framework integrating Kaplan-Meier survival analysis, multivariate Cox proportional hazards regression, logistic regression for recurrence prediction, and machine-learning decision tree classification. This complementary approach—combining traditional parametric models with algorithmic, non-parametric methods—enables the triangulation of evidence and strengthens the reliability of conclusions regarding the prognostic significance of AM in CC.

## METHODS

### Study design and patient population

This retrospective cohort study included patients diagnosed with early-stage CC who were treated at our institution between January 2008 and December 2020. Clinical and pathological data were retrieved from electronic health records. All patients meeting surgical criteria underwent radical hysterectomy with pelvic lymphadenectomy.

Based on final histopathological evaluation, 28 patients with CC and coexistent AM were categorized as the AM (+) group, while 103 patients with CC but without AM were categorized as the AM (-) group. Patients were excluded if they had concurrent primary malignancies, a history of neoadjuvant therapy, or if postoperative pathology confirmed a non-cervical malignancy.

This study was approved by the Institutional Review Board of our hospital (approval number: 2025/010.99/15/28 and date: 30.04.2025) and conducted in accordance with the principles of the Declaration of Helsinki.

### Clinicopathological data collection

The primary objective was to compare clinicopathological characteristics between patients with and without coexistent AM. Variables of interest included tumor size, histological subtype, depth of stromal invasion, presence of lymphovascular space invasion (LVSI), parametrial involvement, infiltration into the vagina or uterine corpus, surgical margin status, and pelvic or para-aortic lymph node metastasis.

Pathological parameters were extracted from final surgical pathology reports, which were independently reviewed by experienced gynecologic pathologists. All pathological evaluations adhered to standardized diagnostic principles in line with the National Comprehensive Cancer Network (NCCN) guidelines^8^. Tumor staging was assigned retrospectively using the International Federation of Gynecology and Obstetrics (FIGO) 2018 classification, which incorporates pathological findings^9^.

### Outcome measures

The secondary objective was to compare OS between the two cohorts. OS was defined as the time interval from histopathological diagnosis to death from any cause or last recorded clinical follow-up. Patients who were alive at last contact were censored at that time point.

### Statistical analysis

Analyses were conducted in R (v.4.4.2). Continuous variables were summarized as means±standard deviation (SD) or medians with interquartile ranges (IQRs) and compared using the independent two-sample t-test or the Mann-Whitney U test, as appropriate. Categorical variables were expressed as counts and percentages, and group differences were assessed using the chi-square test or Fisher's exact test. Survival outcomes were estimated using the Kaplan-Meier method, with group comparisons performed by the log-rank test. Machine learning decision tree classifiers were developed using the rpart package (version 4.1.23) in R (version 4.4.2) to predict recurrence and mortality outcomes. The dataset was partitioned using stratified random sampling with a 70:30 training-to-validation ratio to ensure balanced representation of outcome events, and a fixed random seed (seed=123) was applied for reproducibility. Decision trees were constructed using the classification and regression tree (CART) algorithm with recursive binary splitting. Hyperparameter optimization was performed through 10-fold cross-validation within the training set, with the complexity parameter (cp) evaluated across a grid of values (0.001–0.1) to minimize cross-validated error while preventing overfitting; the optimal cp value was selected based on the minimum x-error criterion, and the tree was pruned accordingly. Additional tree-growing parameters were set as follows: minimum observations per terminal node (minbucket=5), minimum observations required for split attempt (minsplit=20), and the Gini index as the splitting criterion. Given the class imbalance in recurrence outcomes (n=7 recurrence events vs. n=124 non-recurrence), model performance was summarized using metrics robust to imbalanced data, including balanced accuracy, F1-score, and area under the receiver operating characteristic curve (AUC-ROC), in addition to accuracy, sensitivity, specificity, and precision. Model performance was assessed on the held-out validation dataset that was not used during training or hyperparameter tuning; AUC-ROC was computed using the pROC package. Variable importance was quantified using (1) impurity-based importance as reported by rpart (total decrease in Gini impurity attributable to splits on each predictor) and, when applicable, (2) permutation importance computed using the DALEX package by measuring the decrease in validation-set performance after permuting each predictor. These machine learning methods complement traditional regression approaches by capturing complex non-linear relationships and interactions among prognostic variables without requiring a priori specification of functional forms. All statistical analyses were two-sided, and a p<0.05 was considered statistically significant.

## RESULTS

A total of 131 patients with CC were included, of whom 28 (21.4%) had concurrent AM. The two groups were comparable with respect to age and tumor size. However, women with AM had significantly lower gravida (3.7 vs. 4.9, p=0.029) and parity (2.5 vs. 3.5, p=0.022) compared with those without AM. No significant differences were observed in histologic subtype, LVSI, parametrial involvement, lymph node metastasis, or FIGO 2018 stage distribution (Table 1).

**Table 1:** Clinicopathological characteristics, survival outcomes by adenomyosis status.

Characteristic		Adenomyosis present (n=28)	Adenomyosis absent (n=103)	p-value
Age (years), mean±SD		49.9±9.5	49.8±10.5	0.975
Gravida, mean±SD		3.7±2.0	4.9±2.4	0.029
Parity, mean±SD		2.5±1.7	3.5±1.7	0.022
Tumor size (cm), mean±SD		2.1±1.5	2.5±1.7	0.254
Histology, n (%)				
	Squamous cell carcinoma	25 (89.3)	84 (81.6)	0.894
	Adenocarcinoma	3 (10.7)	14 (13.6)	
	Other	0 (0.0)	4 (3.9)	
Depth of stromal invasion, n (%)				
	Superficial <1/3	2 (7.1)	11 (10.7)	0.826
	Middle 1/3	6 (21.4)	19 (18.4)	
	Deep 1/3	20 (71.4)	73 (70.9)	
	Lymphovascular space invasion, n (%)	12 (42.9)	54 (52.4)	0.493
	Vaginal involvement, n (%)	0 (0.0)	4 (3.9)	0.661
	Parametrial invasion, n (%)	1 (3.6)	8 (6.8)	0.876
	Perineural Invasion, n (%)	6 (21.4)	27 (26.2)	0.783
	Pelvic lymph nodes metastasis, n (%)	3 (10.7)	21 (20.4)	0.285
	Para-aortic lymph nodes metastasis, n (%)	0 (0.0)	1 (1.0)	1.000
FIGO stage, n (%)				
	Stage I	24 (85.7)	76 (73.8)	0.384
	Stage II	1 (3.6)	4 (3.9)	
	Stage III	3 (10.7)	23 (22.3)	
	Postoperative adjuvant therapy, n (%)	13 (46.4)	58 (56.3)	0.397
Vital status at last follow-up, n (%)				
	Alive	26 (92.9)	86 (83.5)	0.363
	Deceased	2 (7.1)	17 (16.5)	
	Recurrence, n (%)	0 (0.0)	2 (1.9)	1.000
	Median follow-up (months, IQR)	28.3 (16–57)	36.9 (18–89)	0.218

SD: standard deviation; IQR: interquartile range; FIGO: International Federation of Gynecology and Obstetrics. A p-value of <0.05 indicates a significant difference.

### Survival outcomes

Kaplan-Meier analysis demonstrated significantly shorter OS among patients with AM compared with those without (median OS: 31.3 months [95%CI 17.1–51.1] vs. 64.8 months [95%CI 32.4–78.5]; log-rank p=0.045). At last follow-up, 92.9% of women with AM and 83.5% of those without were alive (Table 2). Recurrence occurred in two patients in the AM (-) group and in none of the AM (+) group.

**Table 2:** Kaplan-Meier overall survival by adenomyosis status.

Group	n	Censored (alive)	Deaths	Median OS (months)	95%CI
Adenomyosis (-)	103	86	17	64.8	32.4–78.5
Adenomyosis (+)	28	26	2	31.3	17.1–51.1

OS: overall survival; CI: confidence interval. Log-rank test χ^2^=4.01, p=0.045.

### Multivariate Cox regression

On Cox regression, age (HR 1.08, 95%CI 1.04–1.13, p<0.001), tumor size (HR 1.48, 95%CI 1.21–1.81, p<0.001), FIGO stage III (HR 5.84, 95%CI 2.20–15.51, p=0.0004), histologic grade G3 (HR 4.19, 95%CI 1.09–16.14, p=0.038), LVSI (HR 4.29, p=0.0004), parametrial infiltration (HR 4.67, p<0.001), and vaginal involvement (HR 24.33, p<0.001) were independently associated with inferior OS. AM did not retain prognostic significance after adjustment (HR 0.91, 95%CI 0.43–1.94, p=0.818) (Table 3).

**Table 3:** Multivariate Cox proportional hazards regression analysis for overall survival.

Variable	Hazard ratio (HR)	95%CI	p-value
Age (years)	1.08	1.04–1.13	<0.001
Tumor size (cm)	1.48	1.21–1.81	<0.001
FIGO Stage III	5.84	2.20–15.51	0.0004
Histologic Grade G3	4.19	1.09–16.14	0.038
LVSI	4.29	1.92–9.56	0.0004
Parametrial infiltration	4.67	2.13–10.21	<0.001
Vaginal infiltration	24.33	3.05–193.99	<0.001
Adenomyosis	0.91	0.43–1.94	0.818
Depth of stromal invasion (mm)	1.01	(0.96–1.07)	0.692
Pelvic LN metastasis	1.68	(0.79–3.56)	0.177

HR: hazard ratio; CI: confidence interval; LVSI: lymphovascular space invasion; FIGO: International Federation of Gynecology and Obstetrics; LN: lymph node. A p-value of <0.05 indicates a significant difference.

The discordance between the univariate Kaplan-Meier analysis and the multivariate Cox regression findings is methodologically instructive and warrants explicit interpretation. While AM demonstrated a statistically significant association with shorter OS in the unadjusted Kaplan-Meier analysis (p=0.045), this apparent prognostic effect was entirely attenuated following adjustment for established clinicopathological determinants in the multivariate Cox model (HR 0.91, p=0.818). This pattern is characteristic of confounding: the univariate association likely reflects the co-distribution of AM with other prognostic variables rather than a true independent prognostic effect. The substantial shift in both the HR (from a significant univariate association to an HR approximating unity) and the p-value (from 0.045 to 0.818) upon multivariate adjustment provides strong evidence that the observed survival difference is attributable to confounding by established risk factors—most notably FIGO stage, tumor size, and LVSI—rather than to an intrinsic prognostic influence of AM itself.

### Predictors of recurrence

Logistic regression analysis identified tumor size (OR 1.95, 95%CI 1.25–3.05, p=0.003) and FIGO stage III disease (OR 29.83, 95%CI 6.51–136.63, p<0.001) as the strongest independent predictors of recurrence. AM showed a trend toward reduced recurrence risk (OR 0.28, 95%CI 0.07–1.18, p=0.083) but did not reach statistical significance.

### Machine learning-based prognostic models

To complement traditional regression models, decision tree classifiers were developed for recurrence and survival prediction. The recurrence model achieved 93.1% overall accuracy, with specificity of 97.0% and sensitivity of 80.0%. Variable importance analysis revealed FIGO Stage 3 (Gini importance 68.6%) and tumor size (20.3%) as the dominant predictors, while AM status contributed minimally (<1%). For OS, the classifier achieved 94.7% accuracy, with age, tumor size, and advanced stage driving the classification. The presence of AM played only a marginal role, acting primarily as a refinement node at terminal branches of the decision tree.

## DISCUSSION

This study provides a comprehensive analysis of the prognostic impact of AM in CC using integrated survival modeling and machine learning approaches. Although univariate analysis revealed shorter OS among patients with AM, this association was substantially attenuated in multivariate Cox regression and decision tree models after adjustment for established prognostic factors, with AM contributing minimally to prognostic classification in both statistical frameworks. These findings indicate that prognostic stratification in CC should remain anchored to established tumor and patient characteristics rather than the presence of concurrent benign uterine pathology.

Our findings indicate that AM in patients with CC does not significantly alter baseline clinicopathological features, treatment patterns, or recurrence rates, suggesting that AM may not inherently induce changes in the tumor's biological behavior, its pathological presentation, or the standard therapeutic approaches undertaken. These observations align with reports on other benign uterine conditions coexisting with gynecologic malignancies. In EC, several studies have reported no significant differences in various clinicopathological features or recurrence rates between patients with and without AM^10,11^, although a comprehensive meta-analysis demonstrated that while AM was associated with improved OS, it did not significantly impact disease-free survival^12^. Similarly, in CC, patients with coexistent AM demonstrated less aggressive tumor behavior, including reduced deep stromal invasion and vaginal infiltration, yet this did not translate into improved survival outcomes^7^. These parallels suggest that for certain benign conditions, their co-presence might not independently drive aggressive tumor characteristics or alter the efficacy of established treatments, particularly when the cancer's intrinsic biology and stage are the primary determinants.

However, Kaplan-Meier analysis revealed that coexisting AM was associated with significantly shorter OS, although this association was not consistently retained in multivariate modeling and machine-learning decision tree analyses once established prognostic factors were accounted for. This phenomenon is frequently encountered in studies investigating the prognostic significance of other benign uterine pathologies. Multiple studies examining AM in EC, for example, have demonstrated an initial survival benefit or association with favorable tumor characteristics in univariate analyses, which subsequently diminished or disappeared entirely in multivariate models after adjustment for established prognostic factors. For instance, Erkilinç et al. reported a significantly higher OS in EC patients with AM in univariate analysis, yet this positive effect was not retained as an independent prognostic factor in multivariate analysis for disease-free survival^13^. Taneichi et al. similarly concluded that despite an association between uterine AM and deep myometrial invasion in stage I endometrioid adenocarcinoma, this did not translate into significant differences in recurrence or mortality rates between groups^14^. The consistent pattern observed across different benign-malignant co-existences underscores the importance of rigorous multivariate analysis to distinguish between independent prognostic factors and those whose apparent impact is mediated by other, more dominant clinical or pathological variables.

Complementing Cox regression with machine learning decision tree analysis strengthened our conclusions through independent validation. Cox models identified independent prognostic factors through HRs, while decision trees ranked variables by their actual contribution to outcome prediction. Both methods concordantly identified FIGO stage, tumor size, and age as the dominant determinants of survival, with AM playing only a marginal role in both frameworks. This cross-validation using fundamentally different analytical approaches—one parametric, one algorithmic—provides robust evidence that AM is not an independent prognostic factor in CC. Moreover, decision trees offer practical clinical utility by generating explicit risk thresholds that can inform point-of-care decision-making, exemplifying modern integration of computational methods into gynecologic oncology practice^15^.

The findings of the present study carry several important clinical implications that merit explicit consideration. First, the demonstration that AM does not constitute an independent prognostic factor in CC after rigorous multivariate and machine-learning adjustment has direct relevance for clinical counseling and treatment planning. Gynecologic oncologists should be reassured that the incidental identification of AM in surgical specimens from CC patients need not prompt modification of treatment strategies, intensification of adjuvant therapy, or alteration of surveillance protocols. Second, the consistent identification of FIGO stage, tumor size, LVSI, parametrial infiltration, vaginal involvement, histologic grade, and age as independent prognostic determinants reinforces the centrality of these established variables in clinical risk stratification. Third, the algorithmically derived decision tree thresholds generated in this study could serve as a complementary tool for clinical decision support, particularly in multidisciplinary tumor board settings where the synthesis of multiple prognostic variables is required to guide individualized treatment planning. These explicit, interpretable risk-stratification rules translate complex statistical models into clinically actionable decision points, facilitating their integration into routine practice.

### Strengths and limitations

This study possesses several notable methodological and conceptual strengths. First, to our knowledge, this is one of the few studies to systematically evaluate the prognostic significance of AM specifically in CC, thereby addressing a meaningful gap in the gynecologic oncology literature where most evidence pertains to EC. Second, the integration of traditional parametric methods with algorithmic machine-learning classification provides a uniquely rigorous, multi-method analytical framework. The concordance of findings across these fundamentally different analytical approaches constitutes methodological triangulation that substantially strengthens the reliability and internal validity of the null finding regarding AM. Third, the machine-learning component incorporated rigorous methodological safeguards against overfitting and biased evaluation, including stratified training-validation partitioning, 10-fold cross-validation for hyperparameter optimization, and reporting of performance metrics robust to class imbalance. Fourth, the comprehensive assessment of established clinicopathological prognostic variables ensured adequate confounder adjustment in the multivariate models, reducing the likelihood that the null finding for AM represents residual confounding. Fifth, the study provides a detailed characterization of both clinicopathological features and survival outcomes, facilitating comparison with future studies and enabling readers to independently assess the comparability of the AM and control cohorts.

Despite these strengths, several limitations should be acknowledged when interpreting the findings of this study. First, the retrospective design inherently carries risks of selection bias and unmeasured confounding that preclude definitive causal inference. Second, the relatively small number of patients with coexistent AM limits statistical power, particularly for detecting modest effect sizes. Adequately powered studies would require substantially larger cohorts to definitively resolve this question. Third, the single-center design may limit external generalizability, as referral patterns, population demographics, surgical techniques, and pathological reporting practices may differ across institutions and geographic settings. Fourth, the absence of systematic data on AM subtypes—including the distinction between focal and diffuse disease, the depth of myometrial penetration, and the degree of glandular activity—represents a notable limitation, as these characteristics may differentially interact with CC biology and could potentially unmask prognostic associations not captured by the binary classification employed in this study. Fifth, the study lacked molecular and immunological data, including HPV genotyping, tumor microenvironment characterization, immune cell infiltration patterns, and molecular profiling, which could elucidate potential biological mechanisms underlying any interaction between AM and CC progression. Sixth, although the median follow-up period was adequate for detecting early survival events, longer follow-up durations may be necessary to capture late recurrences and provide more definitive long-term survival estimates.

## CONCLUSION

Although AM was associated with shorter OS in univariate analysis, it did not function as an independent prognostic factor after rigorous adjustment for established clinicopathological variables through both multivariate Cox regression and machine-learning decision tree modeling. These converging findings across parametric and algorithmic analytical frameworks indicate that prognostic stratification in CC should remain guided by tumor burden, stage, and histopathological risk factors. The incidental identification of AM in surgical specimens should not alter clinical decision-making regarding treatment planning or surveillance protocols.

## Data Availability

The datasets generated and/or analyzed during the current study are available from the corresponding author upon reasonable request.
